# Fluctuating International Normalized Ratio in Patients Compliant on Warfarin: Could Gastroparesis Be the Cause?

**DOI:** 10.7759/cureus.5080

**Published:** 2019-07-04

**Authors:** Mudassar Zia, Nurry Pirani

**Affiliations:** 1 Internal Medicine, University of Missouri Kansas City School of Medicine / Truman Medical Center, Kansas City, USA

**Keywords:** warfarin, mechanical mitral valve, gastroparesis, low molecular weight heparin

## Abstract

Warfarin is the drug of choice to achieve therapeutic anticoagulation in patients with mechanical heart valves. Factors that interfere with the reliable absorption of warfarin may result in difficult to control international normalized ratio (INR) and can cause significant bleeding complications due to supra-therapeutic INR and thromboembolism from sub-therapeutic INR. The patient's non-compliance is an important factor leading to difficult to control INR but there are additional factors that should be considered in difficult cases when dietary and medication compliance are observed. Gastroparesis is one such predominant and overlooked factor.

A 58-year-old African American female with a history of mechanical mitral valve who was on anticoagulation with warfarin was admitted multiple times, with frequent episodes of significant bleeding episodes and fluctuating INR between sub- and supra-therapeutic readings despite being on a relatively stable dose of Coumadin. She was eventually diagnosed with severe gastroparesis, which was the cause of her fluctuating INR.

A case can be made to consider gastric motility testing in such patients, where achieving a therapeutic range for anticoagulation is difficult in the setting of medication and dietary compliance.

## Introduction

Gastroparesis is a well-known complication of long-standing diabetes. A study in Olmsted County, MN, arrived at a cumulative incidence of gastroparesis of 4.8% in type I diabetes, 1% in type 2 diabetes, and 0.1% in the non-diabetic population [1]. Mechanical heart valves carry a high risk of thromboembolism in the absence of therapeutic anticoagulation, with one meta-analysis showing an incidence of four per 100 patient-years, with twice the risk for a mitral valve when compared with the aortic valve [2]. It also carries a risk of valve thrombosis resulting in valve failure and decreased survival, more commonly seen in mechanical valves (95%) located in the mitral position (75%) [3] and with a higher incidence associated with an inadequate level of anticoagulation [3-4].

## Case presentation

A 58-year-old African American female, with a history of mechanical mitral valve placement eight years ago, was admitted multiple times with fluctuating INR despite compliance with diet and warfarin with no change in other medications. Her past medical history was significant for abdominal pain, constipation, chronic obstructive pulmonary disease (COPD), tobacco smoking, hypertension, and obesity.

The patient had been on anticoagulation for several years, with episodes of sub- and supra-therapeutic INRs despite dietary and medication compliance. A cause for these erratic readings could not be found even after several hospital admissions, and the patient continued to have these episodes. Eventually, a hypothesis was proposed that the patient may have erratic absorption of the drug. To confirm this, a gastric emptying study was done, which revealed a gastric emptying time almost nine folds higher than the upper limit of the reference range. In view of this study, A1C was repeated, which came out to be 7.3%. The patient did not have previously diagnosed diabetes mellitus. A gastric outlet obstruction was excluded as a cause of the findings by an esophagogastroduodenoscopy (EGD).

Given the severely prolonged gastric transit time and unpredictable absorption of warfarin, she presented frequently with bleeding from the gastrointestinal (GI) and genitourinary (GU) tract. She also had prolonged hospitalizations for bridging with unfractionated heparin till therapeutic INR was achieved. She did not respond to metoclopramide or erythromycin and with her history of cocaine abuse, domperidone was not attempted due to the risk of arrhythmias. Despite multiple attempts at dietary and medical treatment for gastroparesis and titrating dosage of warfarin, we could not achieve a stable INR within the therapeutic range. Testing for genetic polymorphisms for warfarin was not done. We continued the patient on warfarin with very close outpatient monitoring.

## Discussion

Warfarin has been shown to reduce the risk of thromboembolic events from four per 100 patient years to one per 100 patient years, with an incidence of major bleeding of 1.4 per 100 patient years [2]. A combination of low dose aspirin (81 - 100 mg daily) has shown a further reduction in mortality to 1.4 per 100 patient years albeit with an increased bleeding risk with high-intensity anticoagulation (INR 3-4.5) but not with low-intensity anticoagulation (2.5-3.5) [5-6]. Practice guidelines for anticoagulation for mechanical mitral valve recommend warfarin with a target INR of 2.5-3.5, with low dose aspirin (81-100 mg daily) [7-9].

Factors that interfere with the reliable absorption of warfarin and thereby, resulting in inconsistent, unpredictable, and fluctuating INR, cause significant complications of bleeding from a supra-therapeutic INR and valve thrombosis and failure, as well as thromboembolism from a sub-therapeutic INR, lead us to question if warfarin would be the preferred vehicle of anticoagulation. Gastroparesis is one such predominant factor and, given its prevalence in the diabetic population, it has significant clinical implications. With the delayed gastric emptying time associated with gastroparesis, there is high variability in the absorption of warfarin, leading to an unpredictable INR and the associated aforementioned complications. Therefore, testing for gastric motility should be considered in such cases.

New oral antithrombotic agents are not currently recommended for anticoagulation in patients who had mechanical heart valves after dabigatran was found to have an increased rate of thromboembolic and bleeding events when compared to warfarin, thereby demonstrating an increased risk with no added benefit [10-11].

Our patient had stable INR for about eight years after the placement of a mechanical mitral valve and then started to have difficulty with her INR readings. She had a new diagnosis of diabetes during her admission along with severely prolonged gastric emptying time. Despite close monitoring of warfarin dosing, optimal medical treatment of her gastroparesis, adequate blood glucose control, and control of dietary interferences, it was hard to achieve a stable INR within the therapeutic goal range.

## Conclusions

We are of the opinion that more studies are needed to assess the safety and effectiveness of agents other than Coumadin, including low-molecular-weight heparin (LMWH) in patients where anticoagulation with Coumadin cannot be done reliably. At present, anticoagulation in such cases is anecdotal and data are not robust.

## References

[1] Camilleri M, Bharucha AE, Farrugia G (2011). Epidemiology, mechanisms, and management of diabetic gastroparesis. Clin Gastroenterol Hepatol.

[2] Cannegieter SC, Rosendaal FR, Briët E (1994). Thromboembolic and bleeding complications in patients with mechanical heart valve prostheses. Circulation.

[3] Deviri E, Sareli P, Wisenbaugh T, Cronje SL (1991). Obstruction of mechanical heart valve prostheses: clinical aspects and surgical management. J Am Coll Cardiol.

[4] Dürrleman N, Pellerin M, Bouchard D (2004). Prosthetic valve thrombosis: twenty-year experience at the Montreal Heart Institute. J Thorac Cardiovasc Surg.

[5] Turpie AG, Gent M, Laupacis A (1993). A comparison of aspirin with placebo in patients treated with warfarin after heart-valve replacement. N Engl J Med.

[6] Meschengieser SS, Fondevila CG, Frontroth J, Santarelli MT, Lazzari MA (1997). Low-intensity oral anticoagulation plus low-dose aspirin versus high-intensity oral anticoagulation alone: a randomized trial in patients with mechanical prosthetic heart valves. J Thorac Cardiovasc Surg.

[7] Nishimura RA, Otto CM, Bonow RO (2014). 2014 AHA/ACC guideline for the management of patients with valvular heart disease: executive summary: a report of the American College of Cardiology/American Heart Association Task Force on Practice Guidelines. J Am Coll Cardiol.

[8] Vahanian A, Alfieri O, Andreotti F (2012). Guidelines on the management of valvular heart disease (version 2012): the Joint Task Force on the Management of Valvular Heart Disease of the European Society of Cardiology (ESC) and the European Association for Cardio-Thoracic Surgery (EACTS). Eur Heart J.

[9] Whitlock RP, Sun JC, Fremes SE, Rubens FD, Teoh KH (2012). Antithrombotic and thrombolytic therapy for valvular disease. Antithrombotic Therapy and Prevention of Thrombosis, 9th ed: American College of Chest Physicians Evidence-Based Clinical Practice Guidelines. Chest.

[10] Van de Werf F, Brueckmann M, Connolly SJ (2012). A comparison of dabigatran etexilate with warfarin in patients with mechanical heart valves: The randomized, phase II study to evaluate the safety and pharmacokinetics of oral dabigatran etexilate in patients after heart valve replacement (RE-ALIGN). Am Heart J.

[11] Eikelboom JW, Connolly SJ, Brueckmann M (2013). Dabigatran versus warfarin in patients with mechanical heart valves. N Engl J Med.

